# Gut microbiome composition: link between sports performance and protein absorption?

**DOI:** 10.1080/15502783.2023.2297992

**Published:** 2023-12-27

**Authors:** Péter Fritz, Réka Fritz, Pál Bóday, Ádám Bóday, Emese Bató, Péter Kesserű, Csilla Oláh

**Affiliations:** a Károli Gáspár University of the Reformed Church in Hungary, Faculty of Economics, Health Sciences and Social Studies, Budapest, Hungary; b University of Szeged, Doctoral School of Clinical Medicine, Szeged, Hungary; c Multi-domain Statistics Department, Hungarian Central Statistical Office, Budapest, Hungary; d Cordi R&D nonprofit Inc, Budapest, Hungary; e Xenovea Ltd, Szeged, Hungary; f Eötvös Loránd Research Network, Synthetic and Systems Biology Unit, Institute of Biochemistry, Biological Research Centre, Szeged, Hungary; g University of Pannonia Nagykanizsa - University Center for Circular Economy, Soós Ernő Research and Development Center, Nagykanizsa, Hungary; h University of Duisburg-Essen, Department of Urology, Essen, Germany

**Keywords:** Protein supplementation, microbiome composition, sports performance, athletes, skeletal muscle mass

## Abstract

**Background:**

Sufficient protein intake is essential for adequate physical condition and athletic performance. However, numerous factors can influence the absorption of consumed protein, including timing, type of protein intake, and gut microbiota. In the present study, elite male water polo players consumed a plant-based, vegan protein supplement with (*n* = 10) or without (*n* = 10) pre- and probiotics daily during the 31-day study period.

**Methods:**

We determined the anthropometric characteristics and body composition, dietary habits, gut microbiota composition, and blood parameters of the players at the beginning and at the end of the study. Body composition parameters were analyzed using the InBody 970 bioimpedance analyzer. Gut microbiome composition was determined from stool samples by metagenome sequencing. Paired and unpaired t-tests were used to determine differences between body composition and blood parameters within the groups and between the two groups at the two different sampling times. The Wilcoxon test was used to determine the change in bacterial composition during the study. Correlations between changes in body composition, blood parameters, and taxonomic groups were analyzed using a linear correlation calculation.

**Results:**

Skeletal muscle mass (*p* < 0.001), body cell mass (*p* = 0.002), arm circumference (*p* = 0.003), and protein mass (*p* < 0.001) increased, while body fat mass (*p* = 0.004) decreased significantly in the intervention group which consumed pre- and probiotics in addition to protein supplement. Activated acetate (reductive TCA cycle I) and propionate (pyruvate fermentation to propanoate I) pathways correlated positively with increased skeletal muscle mass (*p* < 0.01 and *p* < 0.05), and the relative abundance of butyrate-producing species showed a significant positive correlation with changes in body fat mass in the intervention group (*p* < 0.05). These correlations were not observed in the control group without the intake of pre- and probiotics.

**Conclusions:**

The composition of the gut microbiota may influence protein absorption and therefore body composition and consequently physical condition and sports performance.

## 1. Background

Sufficient nutrition intake plays an important role in achieving adequate athletic performance. Recognizing this importance, numerous studies have examined the relationship between sports nutrition and players’ adaptation to training intensity and load, recovery time, and thus athletic performance. As a result, guidelines for sport nutrition and a multiplied number of scientific results and publications have become available for elite athletes, nutritional experts, or health-conscious hobby athletes.

Proteins play a prominent role in nutrition, as they are involved in bone and muscle metabolism; and adequate intake thus maintains health status and supports sports performance [1,2]. Serious differences can be observed between the average recommended daily protein requirements of elite athletes and the normal population. It can be stated that athletes generally need twice the daily protein intake. The recommended protein intake for athletes ranges from between 1.4–2.0 g/kg/day [3]. Differences in nutritional strategies can be observed in athletes who primarily engage in endurance or strength training programs, in terms of timing and proportions of daily protein intake. In addition, consumption of other macronutrients may also influence muscle synthesis, making it very difficult to provide general recommendations [4].

A large percentage of consumed proteins are digested by peptidases and pancreatic enzymes, and then absorbed in the small intestine [5]. About 10% of proteins are indigestible and enter the large intestine for further proteolysis by various microbiota [6]. Nowadays, the composition and role of the gut microbiome in relation to sports nutrients have become an increasingly researched area, as it is suggested that the amount and type of protein sources and amino acids determine the diversity of the gut microbiome [7].

Recently, studies have been conducted that clearly demonstrate the link between the effect of high-dose protein intake and possible intestinal inflammation [8], such as inflammatory bowel disease (IBD). Residual undigested proteins can be anaerobically fermented by the microbiota in the hindgut, leading to increased concentrations of ammonia, p-cresol, indole, hydrogen sulfide, histamine, nitric oxide, and biogenic amines, with a higher potential for the intestinal irritations described above [9]. To reduce the risk of possible IBD in humans, an increasing number of protein supplements have appeared on the market in the last decades [10]. Although animal-based whey proteins have been the most popular supplements, plant-based proteins are becoming more popular and could become an alternative for high-quality protein sources. Plant-based proteins have also been shown to provide all essential amino acids, and thus ensuring muscle metabolism [11]. Legumes high in protein and fiber, such as lentils, chickpeas, beans, or peas can be a suitable alternative to animal proteins because they have sufficient protein content compared to other plants. Legumes are the edible seeds or dried grains of plants belonging to the Leguminosae family [12]. In addition, due to their high content of slowly digestible carbohydrates and low fat content, the consumption of legumes decreases the glycemic index and thus reduces the incidence of metabolic diseases [13,14].

In our previous study, we found that elite male water polo players consumed less protein than recommended in the guidelines [15]. In this current study, we conducted a 31-day observational period to examine how a short-term diet of plant-based vegan protein affected muscle development in elite water polo players. The group of players who received the protein metabolism boost also received prebiotics and a fermented probiotic herbal product in addition to the vegan protein supplement (intervention group), while the control group received only the vegan protein supplement. We also investigated possible association between the physical condition of the players and the composition of their gut microbiome after protein supplementation with or without prebiotics and a fermented herbal probiotic.

## 2. Methods

### 2.1. Study design

The study involved 20 elite male water polo players: 3 goalkeepers, 6 wingers, 3 defenders, 5 centers, and 3 shooters. Players were randomly divided into a control group (CON; *n* = 10) and an intervention group (INT; *n* = 10) in an attempt to evenly distribute positions.

Each athlete received 250 ml of Biotech (BioTech USA Kft., Budapest, Hungary) vegan protein shake (concentration was 100 g1^−1^, one 25 g packet dissolved in 250 ml of water) daily during the 31-day study, 30 minutes after the first daily training and 30 minutes before the lunch. The composition of Biotech vegan protein is the following: pea protein, rice protein, soy lecithin, 5.56% L-glutamine, 2.6% L-arginine, 1.6% quinoa flour, 1.1 % goji powder, 0.6% acai powder, NaCl, sucralose, cellulose, carragane. The INT group received a further supplement for 30 days beside the vegan protein at the mornings as: 15 drops of Herbaferm Probiotic solution (Herbaferm Kft., Csömör, Hungary, ingredients: fermented *matricariae* flos, *millefolii* herba, *rosae preudofructus, sambuci* flos, *apis melliferrae* propolis, *querci* cortex, *absinthii* herba, and mixture of naturally occurring *Lactobacillus* strains: *L. buchneri, L. casei, L. kefirii, L. pentosus, L. plantarum, L. brevis, L. rhamnosus, L. paracasei with* 4–5 × 10^6^ CFU). The product was stored at 4 °C. The bacterial composition and CFU of the Herbaferm product remained unchanged until the end of the intervention, which was dissolved in 2 dl of water immediately after waking up. In addition, 3.5 g prebiotic mixture (barley malt, oatmeal, and zeolite) was homogenized daily in their 250 ml vegan protein shake. The protein content of barley and oats given as prebiotics was negligible compared to the daily protein consumption of the players.

**Figure 1.**
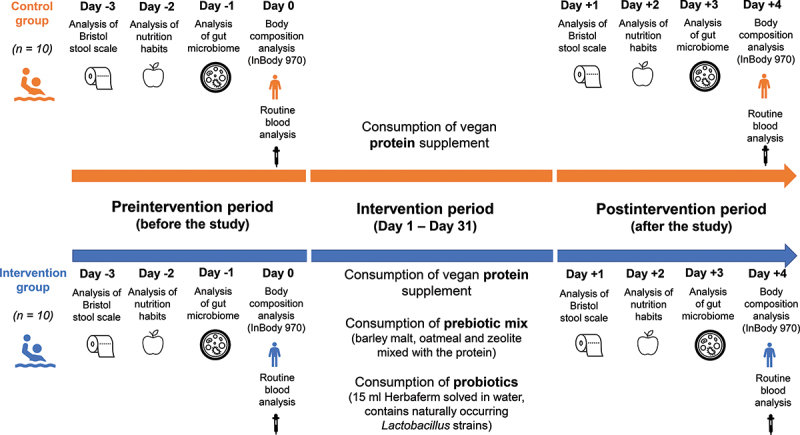
Summary flowchart of the analyses performed during the pre- and postintervention period. During the intervention period, athletes in the control group consumed a vegan protein supplement, while athletes in the intervention group consumed pre-and probiotics in addition to the vegan protein supplement.

The study was performed between 01/10 and 31 October 2021 in Budapest. The study protocol was created in accordance with the Declaration of Helsinki, and it was approved by the Regional/Institutional Science and Research Ethics Committee (BAZ County Central Hospital and University Teaching Hospital (BORS/18/2021). During the 31-day experiment none of the players were under any medical care or got any antibiotics or other treatments that could have influenced the microbiome composition and the result of the study. Figure 1 summarizes the performed analyses and study design.

### 2.2. Analyses and data collections

Anthropometric and body composition analysis was performed with the InBody 970 (InBody Co., Ltd., South Korea). During the InBody measurements, we strictly followed the manufacturer’s recommendations. Participants did not exercise or eat for 3 hours before the test. They did not consume alcohol in the last 24 hours. Before that, they did not go to the sauna, did not take a shower, and did not use body creams or oils. During the analysis, the following parameters were determined: weight (kg), total body water (l), body fat mass (kg), soft lean mass (kg), fat-free mass (kg), skeletal muscle mass (kg), percent body fat (%), visceral fat area (kg), body cell mass (kg), arm circumference (cm), arm muscle circumference (cm), total body water (l), extracellular water (l), intracellular water (l), mineral mass (kg), bone mineral content (kg), protein mass (kg), basal metabolic rate (kcal), and overall fitness score (InBody Score) (https://inbodyusa.com/application/nutrition/).

Blood samples were collected at the start and at the end of the study, before the first training on an empty stomach. After centrifugation (2.500 rpm for 10 minutes), routine blood parameters, as count of red blood cells, value of hemoglobin, hematocrit, mean corpuscular volume (MCV), mean corpuscular hemoglobin (MCH), mean corpuscular hemoglobin concentration (MCHC), red blood cell and platelet distribution width, and vitamin B12 level were determined.

At the beginning and at the end of the study, a self-administered three-day nutrition diary was used to calculate the players’ usual macronutrient intake. Nutrition experts assisted players in accurately completing the nutrition diary. Nutrient and energy intakes were normalized to body weight. Daily energy intake was derived from the caloric value of the meals consumed.

The Bristol stool scale chart was used to determine the stool composition of the players. Stool samples were scored from 1 (severe constipation) to 7 (severe diarrhea) on seven consecutive days before the start of the study and on the last seven days of the study. The scores were then normalized to normal stool composition (score of 4), so that the scores represented deviations from normal composition.

### 2.3. Shotgun metagenome pipeline

For metagenome sequencing, stool samples (~1 g) were collected at the beginning and at the end of the study according to GENIEUR Standard Operating Procedure. Stool samples were stored at −70 °C for 5 days prior the DNA separation. The isolated DNA was stored at −20 °C until sequencing.

For DNA isolation, we used ZymoBIOMICS 96 MagBead DNA kit (Zymo Research) following the manufacturer’s instructions. 100 mg stool sample was lysed in Lysis Buffer with continuous bead beating in ZR Bashing Bead Lysis Tubes for 40 minutes in Vortex Genie 2. After centrifugation, 200 μl supernatant was used for DNA isolation with MagBinding Beads. After the washing steps, DNA was eluted in 50 μl nuclease-free water. DNA concentration was measured by Qubit 3.0 fluorimeter.

Library preparation was performed by Nextflex Rapid XP DNA Seq kit (Perkin Elmer). From each sample, 34 ng DNA was fragmented for 15 minutes. Fragments between 150–300 bp were enriched with double-size selection. After adapter ligation (Nextflex Unique Dual Index barcodes, Perkin Elmer), libraries were amplified for 8 cycles. Library concentration was measured by Qubit HS DNA kit (Thermo Scientific) and fragment size distribution was determined by capillary electrophoresis on Labchip GX Touch Nucleic Acid Analyzer (Perkin Elmer) using DNA NGS 3k Assay kit (Perkin Elmer). The samples were sequenced on NextSeq2000 platform, 2 × 115bp with 30 M reads on average.

The results of metagenome sequencing were analyzed according to the following process: reads were quality trimmed at 3’ and 5’ ends for a minimum of Q30 score, and adapter content, then quality filtered for a minimum average quality score of 30 using FASTQ Toolkit Version: 2.2.5. The trimmed reads were normalized by subsampling to the smallest sample size, for analysis with DRAGEN Metagenomics Pipeline Version: 3.5.11. For host-removal reference version hg38 (alt-aware with HLAs) was used. Results from the DRAGEN Metagenomics Pipeline were combined to a matrix by using the KrakenTools (v1.2) combine_kreports.py script. The resulting table was split by taxonomic groups (phylum, class, ordo, family, genus, species). The quality and adapter trimmed reads (non- subsampled) were the input for the pathway analysis, Humann3 (v3.0.0.alpha.4) was used with CHOCOPhlAn_201901 database, and the EC-filtered uniref90_201901 database for translated search [16]. The forward and reverse read files were merged as recommended by the authors. The results consisted of a gene families table with read per kilobase (RPK) values for each record, and the path abundance table with the calculated raw pathway abundance which were normalized to copies per million (CPM) values using humann_renorm_table script, and the gene families were regrouped to protein families (Pfam) and reactions with the humann_regroup_table script. Reads that were not mapped to either feature in the databases were counted under the label “UNMAPPED.” Similarly mapped reads which could not be integrated into any of the pathways were marked as “UNINTEGRATED.” Each gene family and pathway were also stratified by taxonomy, with the label “unclassified” if no taxonomy could be inferred. Records that could not be regrouped to the new features appeared as “UNGROUPED.” The diversity of microbiome was given by Shannon Diversity Index (H). Shannon Diversity Index was calculated according to Equation: H = -Σpi x ln(pi), where pi meant the proportion of the entire community made up of on species level i, ln: Natural log. The dataset of metagenome sequencing generated and analyzed during the current study are available in the BioProject repository with the following identification number: PRJNA885840 (http://www.ncbi.nlm.nih.gov/bioproject/885840).

### 2.4. Statistical analysis

Paired t-test was used to determine the differences between the body composition variables and the blood parameters measured at baseline and at the end of the study within the groups CON and INT and unpaired t-test was used at the two different sampling times between the two groups. Wilcoxon signed-rank test was used to determine the changes of bacterial amounts between the two groups and within the groups before and after the 31-day study period. Correlations between the anthropometric and body composition characteristics, blood parameters and amounts of different microbial taxonomic groups were performed by using linear correlation models. Raw metagenome sequencing data were evaluated, as it has been described above, then, after normalization, further analyses were performed by using R statistical software packages.

## 3. Results

### 3.1. Changes in anthropometric and body composition parameters

The present study included the adult players (*n* = 20) of a Hungarian elite male water polo team (Ferencváros) with a mean age of 22.7 ± 4.7 years. All participants had long competitive experience in water polo (14.2 ± 4.9 years) with international success, such as World Championships or Olympic Games. During the study period, participants completed 9 dry-land training sessions, 23 in-water training sessions, and 4 games, which meant that they generally trained twice per day, not counting the match days and subsequent rest days.

At the beginning of the study, no significant differences in anthropometric characteristics and body composition were found between the CON and INT groups. At the same time, several changes were observed at the end of the study, especially in the INT group. The only common change during the study was the increased body weight. In the INT group, in addition to the body weight, the parameters of fat-free mass, skeletal muscle mass, body cell mass, arm circumference, arm muscle circumference, protein mass, and InBody score increased significantly. At the same time, the values of body fat mass, percent of body fat and visceral fat area decreased significantly. Therefore, the increased body weight was probably caused by the higher muscle mass. In the CON group, parallelly with the increased body weight, an elevated value of body fat mass was detected (Table 1).

**Table 1.** Anthropometric and body composition characteristics of the control (CON) and intervention (INT) groups at the start and end of the study. Statistical significances were designated with bold characters. Paired t-test is marked by *, while unpaired t-test between the control and intervention groups is marked by **.

Group	Control (*n* = 10)			Intervention (*n* = 10)			Preintervention (CON-1 - INT-1)	Postintervention (CON-2 - INT-2)
Variables	At the start of the study	At the end of the study	P*	At the start of the study	At the end of the study	P*	P**	P**
Weight	92.7 ± 7.2	94.0 ± 7.3	0.005	91.6 ± 13.2	93.5 ± 13.6	0.002	0.810	0.908
Body fat mass	12.9 ± 3.0	13.6 ± 3.4	0.033	13.6 ± 6.1	12.4 ± 5.5	0.004	0.732	0.553
Soft lean mass	75.3 ± 5.7	75.8 ± 5.9	0.150	74.7 ± 8.5	75.5 ± 8.5	0.113	0.862	0.930
Fat-free mass	79.9 ± 6.0	80.4 ± 6.2	0.162	78.9 ± 9.3	80.3 ± 8.9	0.001	0.783	0.982
Skeletal muscle mass	45.8 ± 3.7	46.2 ± 3.7	0.149	45.2 ± 5.4	46.3 ± 5.3	<0.001	0.789	0.977
Percent body fat	13.8 ± 2.8	14.4 ± 3.1	0.081	14.1 ± 4.0	12.9 ± 4.0	<0.001	0.844	0.343
Visceral fat area	60.5 ± 15.9	64.4 ± 17.6	0.051	62.7 ± 31.2	56.5 ± 29.0	0.002	0.843	0.471
Body cell mass	52.5 ± 4.0	52.8 ± 4.0	0.126	52.0 ± 5.9	52.9 ± 5.8	0.002	0.841	0.989
Arm circumference	35.0 ± 1.5	35.2 ± 1.6	0.051	34.4 ± 2.6	34.8 ± 2.6	0.003	0.534	0.687
Arm muscle circumference	32.3 ± 1.3	32.4 ± 1.3	0.378	31.9 ± 2.1	32.2 ± 2.1	0.041	0.545	0.792
Total body water	58.5 ± 4.5	58.9 ± 4.6	0.152	58.0 ± 6.6	58.7 ± 6.6	0.075	0.851	0.947
Intracellular water	36.6 ± 2.8	36.9 ± 2.9	0.116	36.5 ± 4.2	36.7 ± 4.0	0.149	0.867	0.919
Extracellular water	21.8 ± 1.7	22.0 ± 1.7	0.301	21.6 ± 2.4	22.0 ± 2.6	0.073	0.825	0.992
Minerals	5.6 ± 0.4	5.6 ± 0.5	0.229	5.5 ± 0.7	5.5 ± 0.7	0.403	0.714	0.672
Bone mineral content	4.6 ± 0.4	4.7 ± 0.4	0.203	4.5 ± 0.6	4.7 ± 0.5	0.149	0.774	0.828
Protein mass	15.8 ± 1.2	16.0 ± 1.2	0.158	15.7 ± 1.8	16.0 ± 1.7	<0.001	0.840	0.928
Basal metabolic rate	2095.6 ± 131.6	2107.2 ± 133.9	0.150	2080.4 ± 195.5	2099.3 ± 195.6	0.096	0.841	0.918
InBody score	91.3 ± 4.9	91.5 ± 5.4	0.619	87.7 ± 8.1	89.8 ± 7.6	<0.001	0.244	0.572

### 3.2. Daily nutrition intake of participants

We found no significant differences in protein, carbohydrate, fat, and fiber intake between the two groups or between the two different sampling times within the CON and INT groups. In line with these results, nutrition intake was similar between the two groups at the end of the study. None of the athletes fasted during the official season and the study period. In accordance with the nutrition intake, the total energy intake was similar between the two groups at the start and the end of the study. During the research period, no changes could be observed in energy intake within the CON and INT groups (Supplementary Figures S1A and B).

### 3.3. Changes in gut microbiome composition and correlation analysis between body composition parameters and different bacterial strains and metabolic pathways

**Figure 2.**
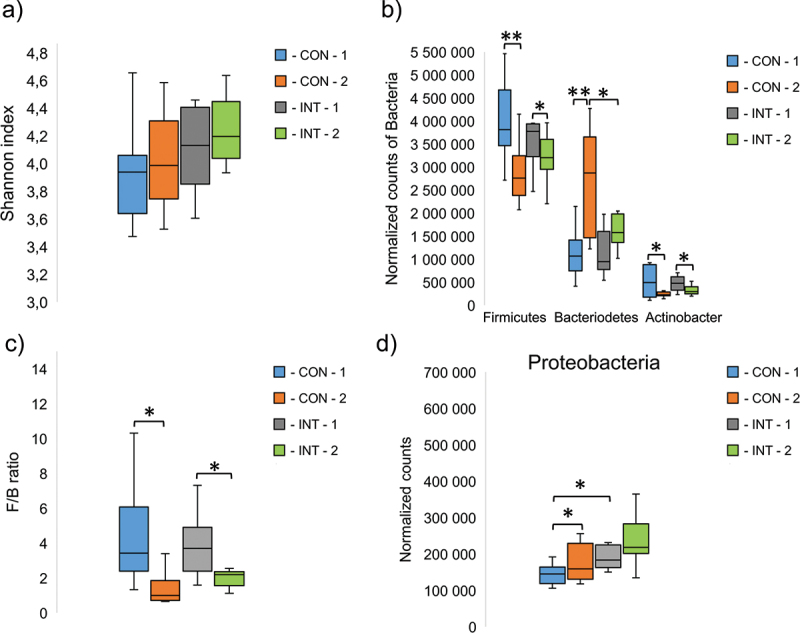
The diversity of intestinal bacterial species, described by the shannon diversity index (a) and the most abundant bacterial phyla (b), Firmicutes/Bacteriodetes ratio (F/B) (c), and normalized counts of proteobacteria phylum (d) CON: control samples, INT: intervention samples. The numbers after the groups represent the different sampling times, 1: at the start of the study, 2: at the end of the study. The significant differences between the groups are signed by **p ≤ 0.01, *p ≤ 0.05.

The analysis of the gut microbiome was performed using the stool samples of the players at the beginning and at the end of the study. The result showed that alpha diversity did not change significantly during the study, regardless of the type of supplement administered; only a slight but non-significant increase was observed in both groups (Figure 2a). Regarding the results at phylum level, *Firmicutes* was the most abundant phylum in both groups at the beginning of the study. During the study period, the relative amounts of *Firmicutes* decreased significantly (CON: *p* < 0.001, INT: *p* = 0.040). The amount of *Bacteroidetes*, the second most abundant phylum, increased significantly in both groups (CON: *p* = 0.006, INT: *p* = 0.053). At the end of the study, a more than two-fold increase was observed in the CON group and a 1.6-fold increase in the INT group, which meant a significant difference between the two groups at the end of the study (*p* = 0.037) (Figure 2b). The *Firmicutes/Bacteroidetes* (F/B) ratio, a commonly used indicator of gut balance [17], also changed significantly in both groups (CON: *p* = 0.012, INT: *p* = 0.040) (Figure 2c).

At the beginning of the study, the *Actinobacteria* phylum was also present in relatively high abundance, however, the relative amount decreased significantly in both groups (CON: *p* = 0.022, INT: *p* = 0.040) (Figure 1b). The relative amount of *Proteobacteria* was also significantly increased in the CON groups compared to the initial bacterial proportion (*p* = 0.033), but not in the INT group (*p* = 0.224). On the other hand, we measured a significant difference between the CON and INT groups at the beginning of the study (*p* = 0.014) (Figure 2d).

To obtain more detailed information on the influence of the gut microbiome on the changes in anthropometric parameters and body composition of players, we aimed to identify some characteristic variations in the composition of gut microbiome at the genus level. We also compared the abundance of indicator strains at the beginning and at the end of the study. However, the relative abundance of the different bacteria showed large individual differences in each case, which made the analysis of the data very complicated.

At the genus level, *Bacteroides, Faecalibacterium*, and *Blautia* were the three most abundant genera in both groups at the end of the study period. After the above genera, *Alistipes*, *Parabacteroides*, *Anaerostipes*, *Bifidobacterium, Anaerobutyricum* in the CON group and the *Roseburia, Collinsella, Prevotella*, *Ruminococcus, Citrobacter, Akkermansia, Lachnospira, Streptococcus* in the INT group were found to be the most presented genera.

During the study period, the highest increase in the relative abundance of *Bacteroides* and *Parabacteroides* was observed. *Odoribacter* was also among the top genera that increased in both groups during the study. The genera *Alistipes* and *Butyricimonas* in the CON, while the genera *Roseburia* and *Paraprevotella* in the INT group were also able to adapt to the changed dietary routine and showed increased amount at the end of the study.

At the species level, some known amounts of acetic, propionic, and butyric acid bacteria were counted. The following bacterial species were observed as acetate producers: *Acetobacterium woodii*, *Akkermansia muciniphila* (also a propionate producer), *Bacteroides thetaiotaomicr*on (also a propionate producer), *Clostridium aceticum*, *Clostridium carboxidivorans*, *Clostridium drakei, Clostridium scatologenes, Clostridium difficile* (also human opportunistic pathogen), *Eubacterium maltosivorans*. Propionate producers were: *Bacteroides fragilis*, *Bacteroides vulgatus*, *Butyricimonas faecalis*, *Prevotella ruminicola*, *Propionibacterium freudenreichii*, *Selenomonas ruminantium*, and the following ones were identified as butyrate producers: *Anaerobutyricum hallii*, *Anaerostipes rhamnosivorans*, *Clostridium butyricum*, *Faecalibacterium prausnitzii*, *Lachnospira eligens, Lachnospiraceae bacterium GAM79*, *Odoribacter splanchnicus* (also a propionate and acetate producer), *Roseburia hominis*, *Roseburia intestinalis, Ruminococcus gnavus* (Supplementary Tables 2 and 3).

**Figure 3.**
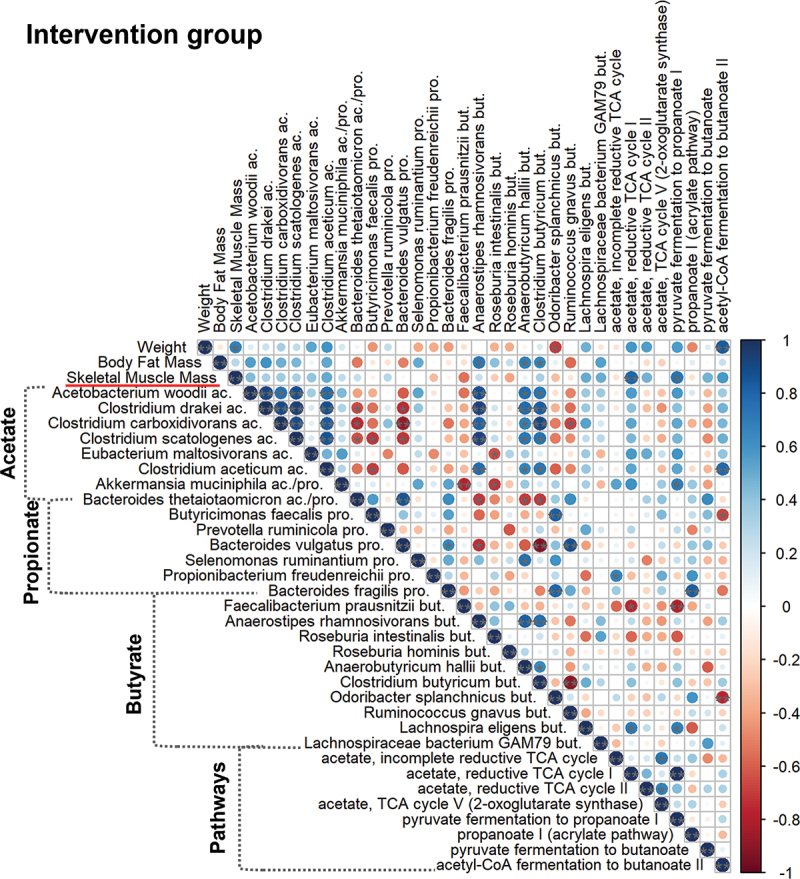
Correlation analysis between weight, body fat mass, skeletal muscle mass and different bacterial strains and metabolic pathways in the intervention group. Pathways for acetate production were the following: incomplete reductive TCA cycle, reductive TCA cycle I, reductive TCA cycle II and TCA cycle V (2-oxoglutarate synthase). Propionate pathways: pyruvate fermentation to propanoate I, propanoate I (acrylate pathway). Pathways for butyrate production were the following: pyruvate fermentation to butanoate, acetyl-CoA fermentation to butanoate II. Statistical significance was designated by **p ≤ 0.01, *p ≤ 0.05.

In the correlation analysis, the changes in the relative abundance of bacteria and the corresponding metabolic pathways were correlated with the changes in weight, body fat mass, and skeletal muscle mass. The results of the analysis revealed a positive correlation between the relative abundance of acetate-producing bacteria and increased skeletal muscle mass in the INT group (Figure 3). In line with this, the reductive TCA cycle I pathway showed a significant positive correlation with skeletal muscle mass in the INT group (*p* < 0.01), and it can be assumed that the presence of these acetate-producing bacteria is responsible for the increased acetate production. Even though pyruvate fermentation to propanoate I and both butyrate biosynthetic pathways showed positive correlations with the increased value of skeletal muscle mass in the INT group, however only the pyruvate fermentation pathway reached the level of significance (*p* < 0.05), and only some propionate (*Prevotella ruminicola*) or butyrate producer bacteria (*Lachnospira eligens, Lachnospiraceae bacterium GAM79*) were identified and fitted to the above-described correlation (Figure 3). No similar correlations were observed in the CON group. In the presence of *Akkermansia muciniphila* (acetate and propionate producer) and in the reductive TCA cycle I pathway even opposite results were observed in the CON group, such as a negative correlation with skeletal muscle mass (*p* < 0.01 and *p* < 0.05, respectively) (Supplementary Figure S2).

For the butyrate-producing species, such as *Anaerobutyricum hallii*, *Anaerostipes rhamnosivorans, and Clostridium butyricum*, the results of the correlation analyses between the changes in body fat mass and the relative abundance of the bacteria showed a significant positive correlation (*p* < 0.05, *p* < 0.05, and *p* < 0.05, respectively). This correlation was found only in the INT group but not in the CON group (Figure 3 and Supplementary Figure S2). In the CON group, the body fat mass increased in 90% of the players, and a positive correlation was found between the body fat mass and propionate-producing bacteria, such as *Akkermansia muciniphila*, *Prevotella ruminicola*, *Propionibacterium freudenreichii* (Supplementary Figure S2).

### 3.4. Changes in blood parameters and correlation analysis between InBody and blood parameters

Continuous training load can lead to changes in laboratory parameters. Therefore, we analyzed numerous blood parameters at the beginning and at the end of the study. We found significant changes in the values of some parameters at the end of the study. However, it is important to note that all laboratory parameters were within the reference range in both groups. Among the laboratory parameters measured, the values of MCH and MCHC showed an elevation in the INT group, but not in the CON group (Supplementary Table S1).

**Figure 4.**
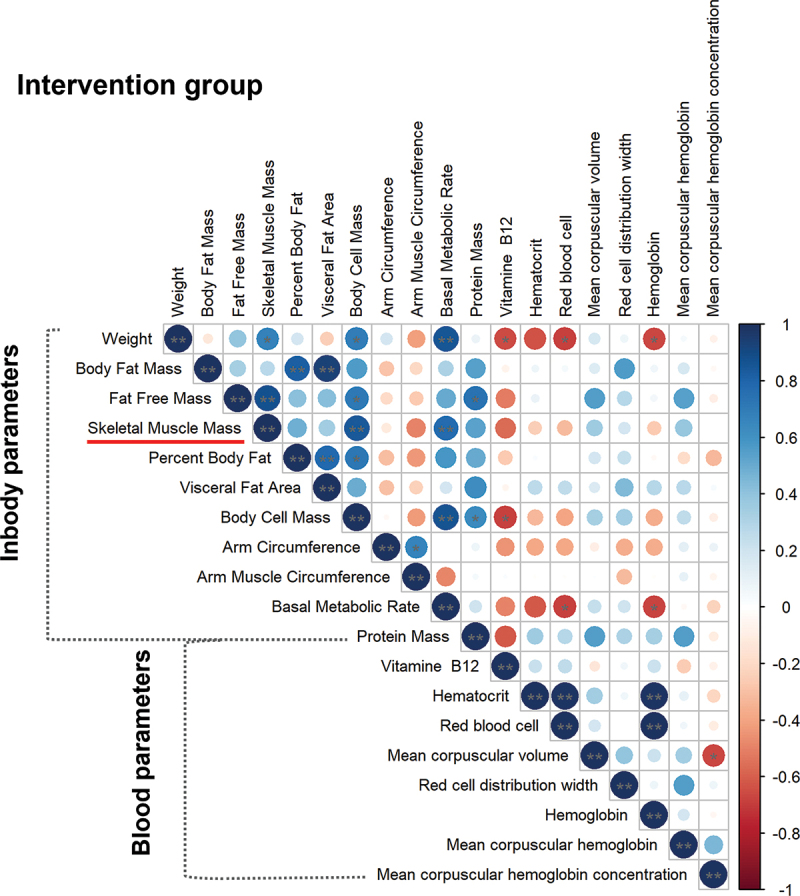
Correlation analysis between InBody and blood parameters in the intervention group. Statistical significance was designated by **p ≤ 0.01, *p ≤ 0.05.

The increased basal metabolic rate, which was characteristic in 50% of CON and in 70% of INT players, showed a significant positive correlation with the change in weight, skeletal muscle mass, and even body cell mass, as well as with the value of fat free mass in the INT group (Figure 4). In the CON group, basal metabolic rate significantly positively correlated with fat-free mass as well as skeletal muscle mass and body cell mass. While increased body fat mass showed a negative correlation with skeletal muscle mass and a positive correlation with percent and visceral body fat and arm circumference in the CON group (Supplementary Figure S3).

Several studies have described the relationship between blood vitamin B12 concentration and hemoglobin, hematocrit, MCV, and MCH. Therefore, vitamin B12 levels are frequently monitored in athletes and administered as a dietary supplement [18,19]. In the present study, we also aim to analyze the relationship between B12 concentration and its possible effects on changes in body composition and laboratory parameters. The change in B12 level correlated positively with hematocrit, the count of red blood cells and hemoglobin in the CON group (Supplementary Figure S3). Interestingly, we did not find these correlations between B12 levels and blood parameters significant in the INT group (Figure 4).

We also analyzed the further correlations between the change in InBody and blood parameters. In the CON group, hemoglobin correlated negatively with fat-free mass, skeletal muscle mass, body cell mass, and basal metabolic rate. MCH showed a negative correlation with red blood cell distribution and skeletal muscle mass (Supplementary Figure S3). Although the values of MCH and MCHC increased significantly in the INT group, they did not show significant correlations with other InBody parameters. Negative correlations were found between weight and the amount of red blood cells, hematocrit, hemoglobin and B12 level. Further negative correlations were found between basal metabolic rate and red blood cell and hemoglobin in the INT group (Figure 3).

### 3.5. Bristol stool test

**Figure 5.**
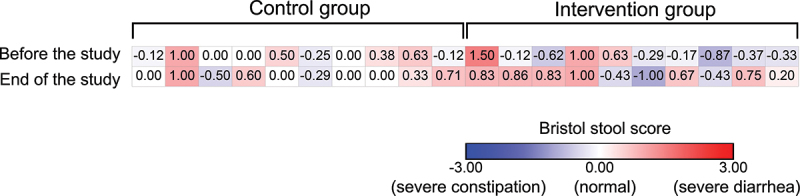
Normalized Bristol stool chart scores of players in the control group (*n* = 10) and intervention group (*n* = 10). The negative values meant constipation (blue), while the positive values meant diarrhea (red).

We analyzed the Bristol scores reported by the players before and at the end of the study. Before the study, the Bristol stool test results showed no significant differences between the CON and INT groups (*p* = 0.065). At the end of the study, players in the INT group had a significant change in stool composition compared to players in the CON group (*p* = 0.029) (Figure 5), but the average stool composition remained in the healthy range.

## 4. Discussion

Researchers are continuously striving to understand the relationship between the gut microbiome and human skeletal muscle axis. Gaining more evidence on the mechanism of how the gut microbiome interacts with human muscle metabolism would be very important in the near future, not only for performance enhancement of elite athletes’, but also for the possible treatment of the age-related decline in muscle mass. In general, athletes need to consume larger amounts of adequate protein to ensure proper muscle biosynthesis and achieve adequate physical condition. However, efficiency depends on several other factors, such as the state of local blood circulation, the type of the muscle training, and the concentration of short-chain fatty acids (SCFA) in the blood; acetate, propionate, and butyrate, which are synthesized by the gut microbiome [20]. All these parameters play a crucial role in the increase of skeletal muscle mass.

We examined the effect of a plant-based vegan protein supplement in combination with pre- and probiotics on anthropometric and body composition characteristics, and thus on the physical condition of elite athletes. In the intervention group, an increase in skeletal muscle mass, body cell mass, arm circumference, arm muscle circumference, and protein mass were observed in parallel with the increase in body weight, whereas a decrease was measured in the values of body fat mass, percent body fat, and visceral fat area at the end of the study. In contrast, increased body fat mass consistent with increased body weight was observed in the control group in which the players consumed only protein supplements. Although the vegan protein supplementation increased body weight in 19 players, skeletal muscle mass increased significantly only in the intervention group. Our results were consistent with the findings of Son et all (2020) [21]. In their study, only the mixture of protein and fiber together were able to enhance muscle synthesis. High quality protein intake after daily training sessions increases muscle protein synthesis. However, it is important to avoid excessive protein intake because the excess protein is converted to fat and stored and can cause hyperlipidemia. As a general principle, protein calorie intake should represent approximately 10% of the body’s total energy requirements [22].

The players, who were divided into the control and intervention groups, received the same diet and had the same training program during the study period. Thus, the difference between the two groups was the additional intake of pre- and probiotics. The key question is mainly the possible relationship between the metabolic processes induced by pro- and prebiotics and the positive changes in body composition parameters.

In general, higher protein consumption indicated changes in the gut microbiome of the players. In the present study, alpha diversity did not change dramatically. Higher protein intake may increase the amount of *Bacteroidetes*, as it has been described by others [23,24]. Lutsiv and colleagues analyzed the changes in microbiome composition induced by various pulse-based protein supplements in mice. Their results show that mice fed peas had one of the most diverse microbial communities. Moreover, they detected a 1.4-fold decrease in the relative abundance of the *Firmicutes* phylum in these mice compared to the control mice [13]. In accordance with their results, we measured a 1.4-fold decrease in the control group and a 1.2-fold decrease in the intervention group. At the same time, a 2.2-fold and a 1.6-fold increase in the amount of *Bacteroidetes* were determined in the control and intervention groups, respectively. The increasing amount of *Bacteroidetes* phylum is consistent with the literature data described above. The changes in phyla *Firmicutes* and *Bacteroidetes* resulted in a 3.1-fold and 1.8-fold decrease in the *Firmicutes/Bacteroidetes* ratio in the control and intervention groups, respectively, which was higher than the results measured in mice [13]. On the other hand, there are several controversial data in the literature on the appropriate *Firmicutes/Bacteroidetes* ratio, which also changes with the age. Vaiserman and colleagues studied in detail the *Firmicutes/Bacteroidetes* ratio in 1550 volunteers. They described a ratio of 0.9 for the age group 20–29, which corresponds to the age of the currently examined players [17]. Biagi and colleagues described that the *Firmicutes/Bacteroidetes* ratio was 3.6 in their study cohort, which included 20 adults aged between 20 and 45 years [25]. At the start of the study, we measured a higher *Firmicutes/Bacteroidetes* ratio with an average of 3.3 and 3.4 in the control and intervention groups, respectively, which decreased to 1.1 and 1.8, probably due to the protein supplementation.

Protein supplementation caused numerous measurable changes in the gut microbiome at the species level. We investigated the possible associations between changes in bacterial composition and dietary supplements. Because of the high-dose protein, the relative abundance of *Odoribacter splanchnicus*, which is known for its butyrate production from lysine [26], increased in each control player but only 60% of the players in the intervention group (Supplementary Tables 2 and 3). The relative abundance of *Roseburia intestinalis* increased in 80% of the players in the intervention group, whereas in only 20% of the control players. The relative abundance of other butyrate-producing bacteria such as *Lachnospira eligens* and *Lachnospiraceae bacterium* GAM79 also changed in both groups. While the abundance of *Lachnospira* and *Lachnospiraceae* increased by 60–70% in the intervention group, only a 30–20% increase was detected in the control group. The species *Roseburia intestinalis, Lachnospira eligens* and *Lachnospiraceae bacterium* GAM79 follow the carbohydrate-pyruvate-Acetyl-CoA pathway to synthesize butyrate [26]. The relative abundance of the known acetic acid bacteria *Clostridium aceticum* changed significantly in both groups. While an increase was observed in 80% of the players in the intervention group, a decrease was observed in 80% of the control players. Although acetate, propionate, and butyrate can be synthesized from amino acids [27], the differences in the changes in these bacterial levels between the two groups can be explained by the presence of the fermentable fibers, which were supplemented only by the players in the intervention group.

It is known that butyrate makes an important contribution to host’ health by providing energy to the intestinal epithelium and modulating the immune system [28], but its role in the gut-muscle axis is not yet clear. Studies by Lv et al. and Barger et al. revealed that higher consumption of dietary fiber resulted in increased numbers *of Faecalibacterium prausnitzii, Lachnospira* sp and, due to higher butyrate production, increased skeletal muscle index in older adults [29,30]. Based on our results, not the *F*. *prausnitzii*, but the overgrow of the bacteria *Lachnospira eligens* and *Lachnospiraceae bacterium* GAM79 might have positively influenced the change in skeletal muscle mass the intervention group (Supplementary Tables 2 and 3). Supplementation of butyrate and propionate improved fat oxidation, insulin sensitivity, and reduced inflammatory processes in young and elderly humans [31,32]. The amounts of monitored propionate bacteria increased in both groups, but a significantly higher occurrence of propionate bacteria was observed only in the control group.

Acetate is generally a regulator of body weight, it affects insulin sensitivity via effects on lipid and glucose homeostasis [33]. Locally in muscle, circulating acetate is used by the cells for ATP generation [34] and it even has some vasodilatory properties [35]. Due to the available prebiotics, not only the amounts of monitored homoacetogenic bacteria but also the corresponding metabolic pathways were increased in the intervention group. The change in acetic acid bacteria showed a positive correlation with the increase in skeletal muscle mass.

According to the available literature, SCFA affect skeletal muscle mass more under conditions of metabolic stress or increased metabolic demand [36], which may be occurred in the case of players in their intense training period. The regulatory effect of SCFA on host homeostasis largely depends on their concentrations, but their proper ratio in the circulation may be even more important.

## 5. Conclusions

Vegan protein supplementation improved body weight in 19 players, but skeletal muscle mass increased significantly only in the intervention group. We hypothesize that due to the additional intake of prebiotics and probiotics, fermentation of SCFA by the gut microbiome was more efficient in the intervention group, which may have contributed to skeletal muscle development. The increased muscle metabolism could be supported by the elevated MCH and MCHC levels in the blood, which may improve the metabolic activities of muscle cells. The positions of the players (goalkeeper, wing, defender, center, shooter) showed no association with the level of muscle metabolism. Further studies with a larger number of participants are needed to gain a deeper understanding of the effects of protein and symbiotic supplements on muscle biosynthesis and overall homeostasis in elite athletes.

## Supplementary Material

Supplemental Material

## Data Availability

All data generated and analyzed during the current study are included in this published article [and its supplementary information file]. The dataset of metagenome sequencing generated during the current study are available in the BioProject repository (http://www.ncbi.nlm.nih.gov/bioproject/885840).

## Abbreviations

Con: control group
Intervention: intervention group
MCH: mean corpuscular hemoglobin
MCHC: mean corpuscular hemoglobin concentration
SFCA: short chain fatty acid
