# Supplementary material for: Gut microbiome composition: link between sports performance and protein absorption?
Source: J Int Soc Sports Nutr. 2023 Dec 27;21(1):2297992. doi: 10.1080/15502783.2023.2297992 (PMC10763846; doi:10.1080/15502783.2023.2297992)
Supplement: Supplemental Material [file RSSN_A_2297992_SM8835.docx]

**Supplementary Material**

*Figures*


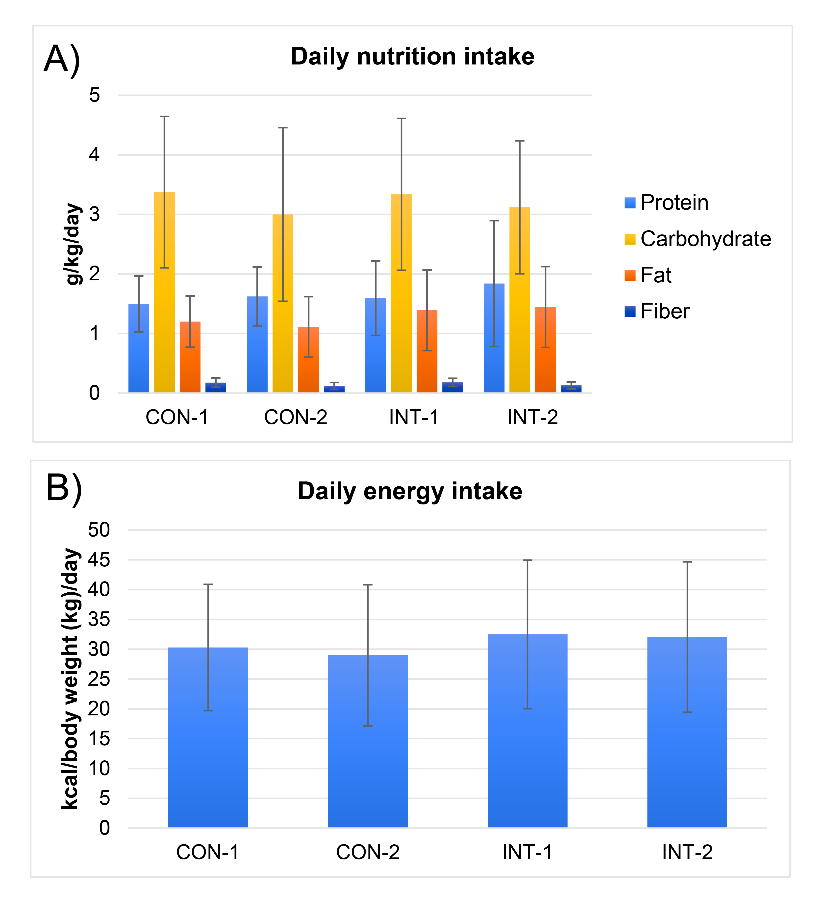


**Supplementary Figure 1.** Daily macronutrient (A) and energy (B) intake of the players in the control (CON) and intervention (INT) groups at the beginning (1) and the end of the study (2). Both groups included 10-10 players.


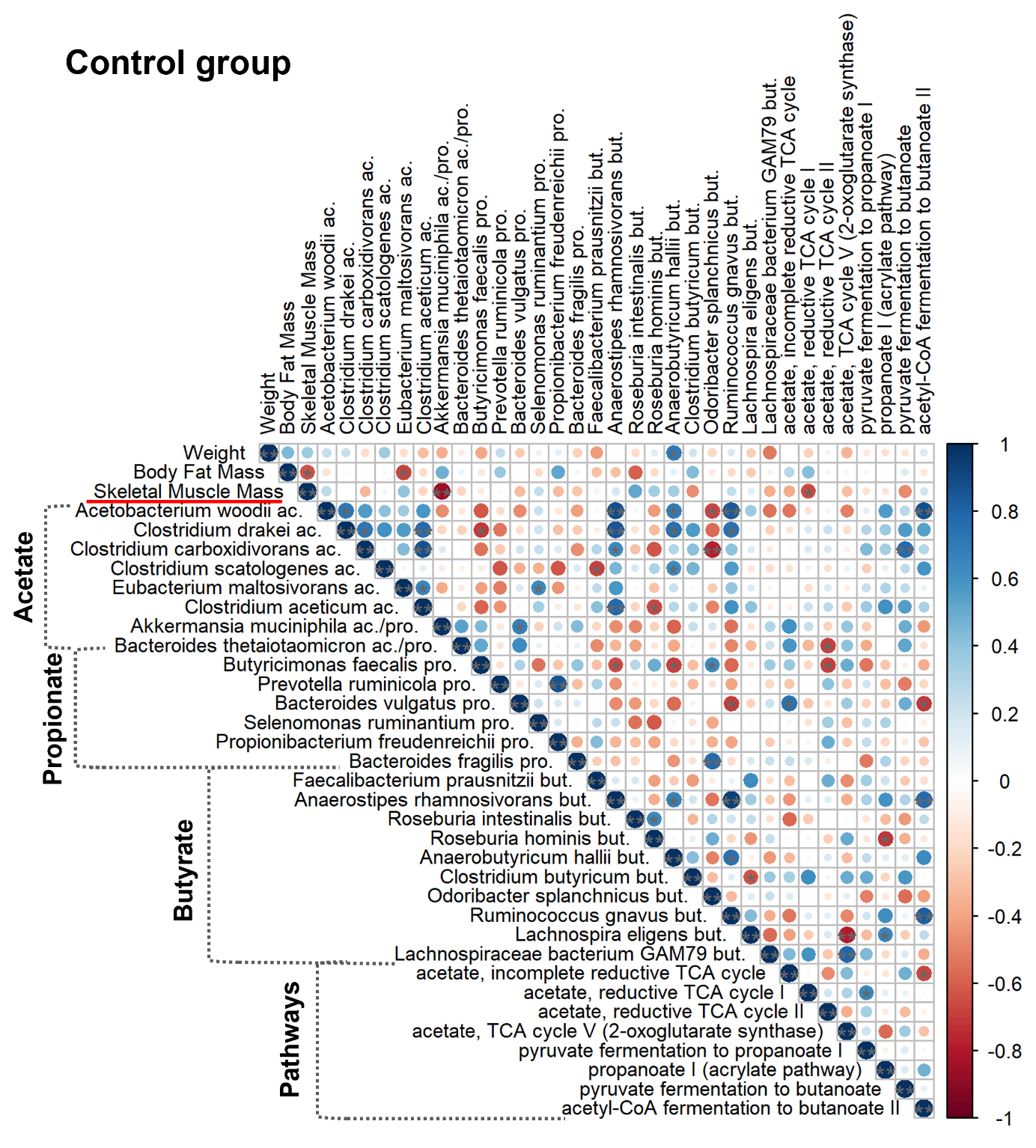


**Supplementary Figure 2.** Correlation analysis between weight, body fat mass, skeletal muscle mass and various bacteria strains and metabolic pathways in the control group. Pathways for acetate production were the following: incomplete reductive TCA cycle, reductive TCA cycle I, reductive TCA cycle II and TCA cycle V (2-oxoglutarate synthase). Propionate pathways: pyruvate fermentation to propanoate I, propanoate I (acrylate pathway). Pathways for butyrate production were the following: pyruvate fermentation to butanoate, acetyl-CoA fermentation to butanoate II. Statistical significance was designated by ** p≤0.01, * p≤ 0.05.


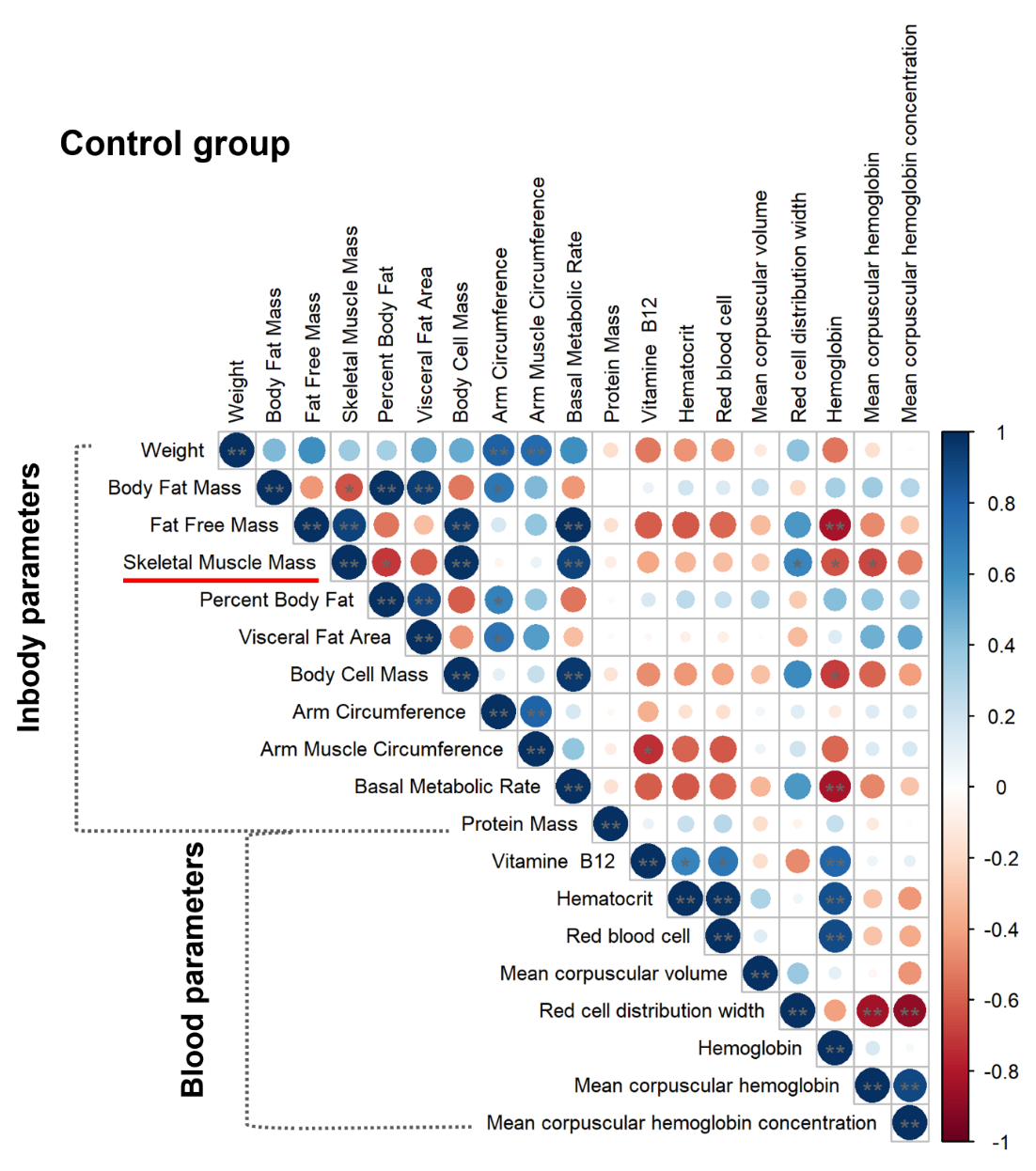


**Supplementary Figure 3**. Correlation analysis between InBody and blood parameters in the control group. Statistical significance was designated by ** p≤0.01, * p≤ 0.05.

*Table*

**Supplementary Table 1.** Changes in the values of measured blood parameters during the 31-day research period in the Control and Intervention groups. Statistical significances were designated with bold characters. Paired t-test is marked by *, while unpaired t-test between the control and intervention groups by **. RBC: red blood cell, RWD-CV: Red blood cell Distribution Width, MCV: mean corpuscular volume, HCT: hematocrit, HGB: hemoglobin, MCH: mean corpuscular hemoglobin, MCHC: mean corpuscular hemoglobin volume.

**Supplementary Table 2.** Changes in the relative abundance of some short-chain fatty acid (SCFA)-producing bacteria in the control group during the 31-day research period. *= acetate- and propionate-producing strain. ** strain is also human opportunistic pathogen.

| **Control group (n=10)** | | | | | | | | | | | |
| --- | --- | --- | --- | --- | --- | --- | --- | --- | --- | --- | --- |
| **SCFA** | **Bacteria species** | **Changes in relative abundance (%)** | | | | | | | | | |
|  |  | **1.** | **2.** | **3.** | **4.** | **5.** | **6.** | **7.** | **8.** | **9.** | **10.** |
| **Acetate** | *Acetobacterium woodii* | -54.6 | -11.4 | -11.9 | -53.4 | -45.8 | 9.1 | 38.4 | -45.2 | -39.4 | -53.5 |
|  | *Akkermansia muciniphila** | 165.6 | -73.7 | -57.8 | -45.2 | -15.3 | -83.6 | 24.6 | -12.7 | -68.8 | 185.8 |
|  | *Bacteroides thetaiotaomicron** | 708.4 | 56.9 | 101.6 | 333.5 | 352.0 | 226.7 | 131.0 | 155.2 | 259.0 | 3719.7 |
|  | *Clostridium aceticum* | -63.0 | -21.4 | -25.5 | -49.0 | -49.4 | -9.7 | 79.3 | -29.5 | 16.4 | -21.8 |
|  | *Clostridium carboxidivorans* | -45.2 | -9.6 | -33.5 | -43.8 | -43.2 | 1.6 | 67.8 | -49.3 | -4.9 | -25.9 |
|  | *Clostridium difficile*** | -37.2 | -15.7 | -14.2 | -56.7 | -43.7 | -20.3 | -1.1 | -31.2 | -16.5 | -48.7 |
|  | *Clostridium drakei* | -64.2 | 11.0 | -39.9 | -69.3 | -56.1 | 6.2 | 5.0 | -47.3 | -20.8 | -43.5 |
|  | *Clostridium scatologenes* | -61.9 | 10.9 | -23.1 | -63.2 | 5.0 | 29.2 | -28.6 | 0.0 | -39.9 | -35.1 |
|  | *Eubacterium maltosivorans* | -35.1 | -24.4 | 7.3 | -55.6 | -51.1 | -26.6 | -13.9 | -45.4 | -5.5 | -26.6 |
| **Propionate** | *Bacteroides fragilis* | 253.6 | 37.4 | 85.1 | 73.9 | 205.7 | 38.3 | 47.4 | 324.3 | 199.3 | 996.8 |
|  | *Bacteroides vulgatus* | 348.3 | 23.0 | -5.2 | 94.0 | 15.0 | 33.7 | 65.0 | 277.7 | 825.7 | 698.2 |
|  | *Butyricimonas faecalis* | 364.6 | -33.3 | 61.3 | 205.4 | 718.3 | 76.2 | 103.4 | 1308.8 | 163.5 | 2533.1 |
|  | *Prevotella ruminicola* | 93.7 | 946.5 | 68.1 | 2214.8 | 121.5 | 40.1 | -13.7 | 172.0 | 214.5 | 477.0 |
|  | *Propionibacterium freudenreichii* | 4.2 | 52.0 | -7.9 | 139.7 | -11.7 | 9.9 | 130.7 | -39.7 | 6.0 | 85.7 |
|  | *Selenomonas ruminantium* | -19.4 | -31.6 | 16.9 | -14.7 | -71.7 | 3.7 | 13.0 | -19.8 | 10.0 | -19.7 |
| **Butyrate** | *Anaerobutyricum hallii* | -49.6 | 70.4 | 2.7 | -47.4 | -27.7 | 10.8 | -12.5 | -29.2 | -17.9 | -30.2 |
|  | *Anaerostipes rhamnosivorans* | -56.1 | -11.2 | -13.4 | -50.9 | -38.0 | -3.2 | 29.0 | -30.9 | -10.6 | -48.7 |
|  | *Clostridium butyricum* | -55.1 | 7.8 | -59.2 | -55.8 | -40.4 | -3.8 | -40.1 | -46.5 | 1.3 | -24.4 |
|  | *Faecalibacterium prausnitzii* | -56.1 | -35.8 | -6.6 | -0.8 | -41.1 | -37.9 | 16.4 | -30.1 | -7.6 | -26.7 |
|  | *Lachnospira eligens* | -55.9 | -28.8 | 32.2 | -15.4 | -8.2 | -72.3 | 54.6 | 44.7 | -33.2 | -32.8 |
|  | *Lachnospiraceae bacterium* | -9.6 | -62.1 | -41.9 | -51.7 | -39.2 | -17.3 | -56.3 | -25.9 | 258.1 | 9.9 |
|  | *Odoribacter splanchnicus* | 151.2 | 67.8 | 137.6 | 105.2 | 241.7 | 13.7 | 40.6 | 1128.4 | 303.1 | 4291.4 |
|  | *Ruminococcus gnavus* | -69.8 | 10.8 | -10.5 | -64.8 | -27.5 | -21.0 | 33.5 | -25.3 | -17.8 | -56.0 |
|  | *Roseburia hominis* | -31.6 | -11.2 | 39.7 | -55.4 | 101.3 | -23.4 | -74.9 | -28.1 | -12.3 | 1.7 |
|  | *Roseburia intestinalis* | -62.0 | -25.4 | 251.7 | -54.6 | 184.5 | -17.1 | -30.4 | -34.5 | -28.2 | -29.5 |

**Supplementary Table 3.** Changes in the relative abundance of some short-chain fatty acid (SCFA)-producing bacteria in the intervention group during the 31-day research period. *= acetate- and propionate-producing strain. ** strain is also human opportunistic pathogen.

| **Intervention group (n=10)** | | | | | | | | | | | |
| --- | --- | --- | --- | --- | --- | --- | --- | --- | --- | --- | --- |
| **SCFA** | **Bacteria species** | **Changes in relative abundance (%)** | | | | | | | | | |
|  |  | **1.** | **2.** | **3.** | **4.** | **5.** | **6.** | **7.** | **8.** | **9.** | **10.** |
| **Acetate** | *Acetobacterium woodii* | -47.8 | -17.6 | 52.8 | -10.8 | -5.8 | -19.7 | 23.3 | -43.8 | -25.1 | -18.7 |
|  | *Akkermansia muciniphila** | 145.6 | -80.4 | 219.4 | 43.1 | 26.4 | -7.0 | -80.8 | -84.5 | 4836.1 | -5.1 |
|  | *Bacteroides thetaiotaomicron** | 1285.6 | -41.2 | -17.4 | 55.7 | 71.4 | 209.9 | 42.7 | -20.4 | 75.1 | 75.1 |
|  | *Clostridium aceticum* | -29.7 | 9.7 | 88.8 | 2.0 | 20.1 | 26.2 | 49.2 | 30.3 | 3.7 | -30.9 |
|  | *Clostridium carboxidivorans* | -82.5 | -10.0 | 73.7 | -36.6 | 11.3 | -20.2 | 79.9 | 84.2 | -41.2 | -32.7 |
|  | *Clostridium difficile*** | -38.9 | 13.6 | 25.5 | -4.2 | 7.2 | -45.9 | -8.9 | -14.0 | -16.4 | -41.3 |
|  | *Clostridium drakei* | -65.1 | 5.2 | 145.2 | -36.8 | 61.2 | -37.9 | 85.8 | -30.4 | -19.7 | -34.4 |
|  | *Clostridium scatologenes* | -71.6 | 9.3 | 74.8 | -27.6 | 5.2 | -16.0 | 29.0 | -7.7 | -51.5 | -29.9 |
|  | *Eubacterium maltosivorans* | -22.4 | -20.8 | 0.3 | 11.9 | -21.7 | 3.1 | -3.8 | -22.8 | -5.6 | -23.9 |
| **Propionate** | *Bacteroides fragilis* | 567.7 | 0.7 | 113.0 | 112.7 | 41.3 | 46.8 | 16.5 | -25.7 | 128.8 | 226.3 |
|  | *Bacteroides vulgatus* | 456.3 | 15.1 | 64.7 | 213.3 | 11.6 | 237.7 | -4.4 | -21.9 | 123.8 | 344.4 |
|  | *Butyricimonas faecalis* | 607.3 | -19.1 | -33.2 | 34.7 | 9.7 | 52.1 | 12.2 | -23.7 | 133.3 | 364.1 |
|  | *Prevotella ruminicola* | 133.2 | 26.9 | -20.8 | -78.7 | 92.9 | 48.4 | 70.0 | 320.9 | -41.7 | 2842.3 |
|  | *Propionibacterium freudenreichii* | -60.9 | 2.6 | 31.6 | -69.1 | 1.3 | 0.1 | -82.7 | -69.7 | -17.4 | 121.6 |
|  | *Selenomonas ruminantium* | -20.6 | -21.2 | 7.6 | -5.7 | -25.3 | -31.4 | -5.0 | -32.2 | 1.2 | -7.4 |
| **Butyrate** | *Anaerobutyricum hallii* | -51.8 | -13.5 | 46.0 | 5.3 | -44.0 | -66.1 | 14.5 | -31.6 | -11.0 | -36.1 |
|  | *Anaerostipes rhamnosivorans* | -47.1 | 11.3 | 39.2 | -13.7 | -15.1 | -21.9 | 7.9 | -30.4 | -21.2 | -16.0 |
|  | *Clostridium butyricum* | -88.6 | -22.2 | 125.9 | -48.1 | 13.5 | -7.0 | 87.9 | 40.3 | -10.4 | -27.7 |
|  | *Faecalibacterium prausnitzii* | 0.1 | 30.1 | -55.6 | 7.1 | 0.7 | -24.0 | -13.0 | -11.9 | -17.1 | -23.9 |
|  | *Lachnospira eligens* | -47.9 | -70.9 | 7.4 | -36.3 | 26.5 | -28.1 | 188.7 | 129.2 | 27.2 | 14.1 |
|  | *Lachnospiraceae bacterium* | -29.5 | 125.7 | 66.6 | -22.7 | 2.3 | 105.7 | 152.8 | -54.0 | 2.6 | 31.8 |
|  | *Odoribacter splanchnicus* | 1558.0 | -8.7 | 62.6 | 108.3 | 9.4 | -25.4 | -19.2 | -58.7 | 132.9 | 442.7 |
|  | *Ruminococcus gnavus* | 385.2 | 30.4 | 35.7 | -11.3 | -18.1 | -35.1 | -6.8 | -19.2 | -23.6 | -0.8 |
|  | *Roseburia hominis* | -37.2 | 23.4 | -31.5 | 113.5 | -24.3 | -18.2 | 8.7 | -15.0 | 22.4 | -5.4 |
|  | *Roseburia intestinalis* | -18.4 | 273.7 | 31.9 | 5.6 | 81.9 | 22.8 | 10.8 | 6.5 | -8.9 | 54.5 |
